# Age-related changes of intraocular pressure in Dutch belted rabbits

**DOI:** 10.1038/s41598-024-62097-w

**Published:** 2024-05-27

**Authors:** Young In Shin, Young Kook Kim, Jin Wook Jeoung, Ki Ho Park

**Affiliations:** 1 Department of Ophthalmology, Seoul National University College of Medicine, Seoul, 03080 South Korea; 2 Department of Ophthalmology, Gachon University Gil Hospital, Incheon, South Korea; 3 Department of Ophthalmology, Seoul National University Hospital, Seoul, South Korea

**Keywords:** Ophthalmology, Ophthalmic tests, Intraocular pressure, Glaucoma, Tonometry, Dutch belted rabbits, Animal physiology, Ocular hypertension

## Abstract

This study investigated intraocular pressure (IOP) in Dutch belted rabbits using two different tonometers, rebound tonometry (TonoVet Plus; TVP) and a Tonopen (Tono-Pen AVIA Vet; TPA). Post-pubescent male Dutch belted rabbits aged 36 weeks (n = 10 animals) were used in the study. IOP measurements were conducted every 2 weeks for 22 weeks using TVP and TPA on both eyes of each rabbit. The average IOP measurements were compared by the paired Student’s t-test. Pairwise Pearson’s correlation coefficients and Bland–Altman statistics were used. The overall mean IOP measured with TPA was significantly higher than that with TVP (23.5 ± 4.9 vs. 21.8 ± 2.4 mmHg for the right eyes; *P* = 0.045, and 23.0 ± 4.7 vs. 21.5 ± 2.4 mmHg for the left eyes; *P* = 0.047). Both tonometers tended to show increased IOP readings with age, and positive correlations between IOP and age were observed with both TPA (r = 0.95, *P* < 0.001 for right eyes; r = 0.95, *P* < 0.001 for left eyes) and TVP (r = 0.91, *P* < 0.001 for right eyes; r = 0.64, P = 0.024 for left eyes). The average bias calculated by subtracting TPA from TVP was − 1.60 (95% confidence intervals − 1.927, − 1.281) mmHg. IOP in post-pubescent Dutch belted rabbits tended to increase with age throughout the 22 week study.

## Introduction

Intraocular pressure (IOP) is maintained by a delicate balance between production and drainage of aqueous humor. Glaucoma, a progressive optic neuropathy that is caused by IOP dysregulation that can result in irreversible vision loss. Therefore, understanding the factors that influence IOP and its regulation is essential to any full understanding of glaucoma. Investigations on IOP are carried out with a variety of approaches, including experimental studies using both human and animal subjects.

Because of the similarities between rabbits’ ocular anatomy and physiology and those of humans’, rabbits are frequently employed as animal models for research on IOP and glaucoma^1,2^. Dutch belted rabbits in particular are favored for ocular studies, given their pigmented irides. Dutch belted rabbits’ IOP is higher in post puberty than in pre puberty, according to Hays et al.^3^. This might be owed to the fact that when rabbits age, the cornea thickens, the episcleral venous pressure increases, and the aqueous outflow slows^4,5^. On the age-related changes in the IOP of Dutch belted rabbits, however, there is little information. Such knowledge nonetheless is crucial for accurate interpretation of experimental findings, establishment of reference ranges, and design of effective therapeutic strategies in both animal research and human medicine. Accordingly, in this study, we investigated the age-related changes of IOP in pot-pubescent Dutch belted rabbits and explored the possible mechanisms behind them.

## Results

For each eye of ten Dutch belted rabbits, 360 readings from TonoVet Plus (TVP) and 360 from Tono-Pen AVIA Vet (TPA) were gathered for the IOP measurements, giving a total of 720 readings per tonometer. The IOP data was symmetrically distributed around the mean. The overall mean IOP was higher when measured by TPA than by TVP when comparing the two tonometer readings for the same eye (23.5 ± 4.9 vs. 21.8 ± 2.4 mmHg for the right eyes; *P* = 0.045, and 23.0 ± 4.7 vs. 21.5 ± 2.4 mmHg for the left eyes; *P* = 0.047, Table 1). The average IOP obtained by both tonometers in the right and left eyes did not differ statistically (*P* > 0.05). Table 2 demonstrates the IOP values measured with two different tonometers at all times during 22 weeks of follow-up period. All of the IOP profiles ranged from 15.0 to 30.0 mmHg by TPA and from 14.0 to 28.0 mmHg by TVP.

**Table 1:** Mean values and standard deviation of intraocular pressure in Dutch belted rabbits (n = 10 animals) measured with TonoVet Plus and Tono-Pen AVIA Vet.

Tonometer	Laterality	Total readings	Total mean ± standard deviation
TonoVet Plus	Right eye	210	21.8 ± 2.4^b^
	Left eye	210	21.5 ± 2.4^b^
Tono-Pen AVIA Vet	Right eye	210	23.5 ± 4.9^a^
	Left eye	210	23.0 ± 4.7^a^

Values are mean ± standard deviation.

Statistically significant variations are indicated by different superscript letters (*P* < 0.05).

**Table 2:** Age-related change of intraocular pressure measured by rebound tonometer (TonoVet Plus) and Tonopen (Tono-Pen AVIA Vet).

Tonometer	Laterality	Age (weeks)											
		36	38	40	42	44	46	48	50	52	54	56	58
TonoVet Plus	Right eye	19.0 ± 2.3	20.3 ± 2.9	20.5 ± 1.5	20.9 ± 2.2	21.7 ± 1.8	21.0 ± 2.0	21.9 ± 2.1	21.7 ± 1.2	22.5 ± 2.5	22.0 ± 1.9	22.3 ± 3.1	22.5 ± 2.1
	Left eye	20.8 ± 2.1	20.3 ± 2.8	20.6 ± 2.1	21.7 ± 1.7	21.9 ± 2.9	21.0 ± 2.6	21.5 ± 1.8	21.0 ± 1.9	22.6 ± 1.2	21.2 ± 1.4	21.5 ± 1.2	22.6 ± 1.6
Tono-Pen AVIA Vet	Right eye	19.6 ± 5.1	21.3 ± 4.5	22.7 ± 4.4	22.5 ± 2.9	23.4 ± 4.2	23.2 ± 5.8	23.7 ± 4.6	23.5 ± 4.5	23.7 ± 4.9	23.9 ± 5.2	24.4 ± 6.2	24.9 ± 5.1
	Left eye	21.5 ± 4.1	21.7 ± 2.9	21.9 ± 2.9	22.0 ± 2.9	22.9 ± 3.2	23.9 ± 2.4	23.2 ± 5.2	23.5 ± 3.6	23.8 ± 2.8	23.8 ± 4.6	24.5 ± 5.1	24.5 ± 5.2

Values are mean ± standard deviation.

**Figure 1:**
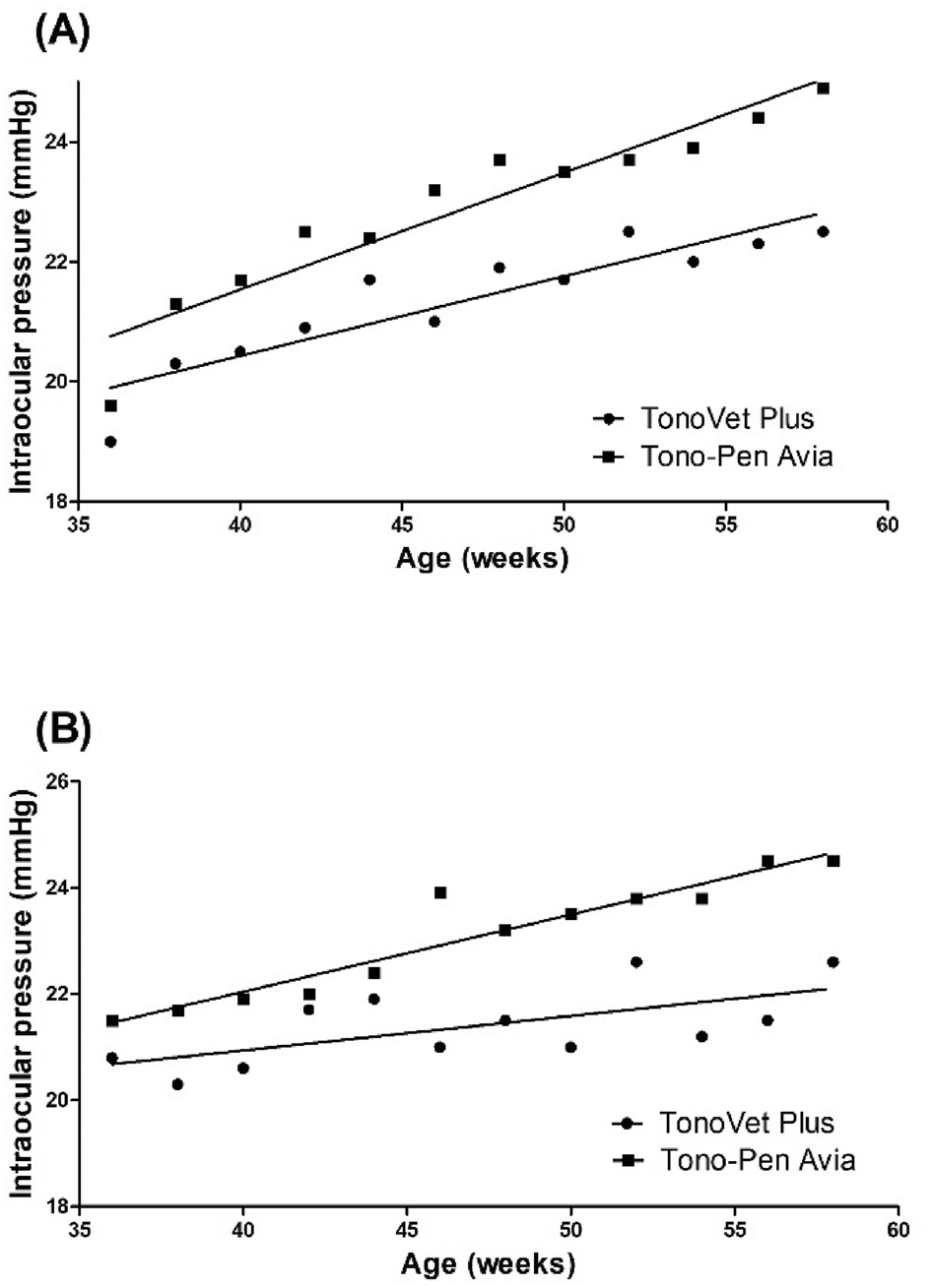
Scatterplots and linear regression models showing average intraocular pressure (IOP) profiles of post-pubescent Dutch belted rabbits during 22 weeks of follow-up. Both the Tono-Pen AVIA Vet (TPA) and TonoVet Plus (TVP) detected age-related increases in IOP in both eyes, and TPA readings were consistently higher than the rebound tonometry results during the entire study period (*P* = 0.023 for right eyes (**A**), *P* = 0.031 for left eyes (**B**) by repeated-measures ANOVA test). IOP measurements between 36 and 58 weeks of age showed consistent age correlations with both the TPA and TVP ((**A**) right eyes, r = 0.95, *P* < 0.001 for TPA, r = 0.91, *P* < 0.001 for TVP; (**B**) left eyes, r = 0.95, *P* < 0.001 for TPA, r = 0.64, P = 0.024 for TVP).

Figure 1 shows the IOP profiles of the Dutch belted rabbits during the 22 week study period. Age-related increases in IOP were observed with both tonometers for each eye, and throughout the entire period, TPA readings were continuously greater than TVP values (*P* = 0.023 for right eyes, *P* = 0.031 for left eyes). The IOP values for 36 to 58 weeks of age were positively correlated with age, both with TPA (r = 0.95, *P* < 0.001 for right eyes; Fig. 1A   and r = 0.95, *P* < 0.001 for left eyes; Fig. 1B) and TVP (r = 0.91, *P* < 0.001 for right eyes; Fig. 1A  and r = 0.64, P = 0.024 for left eyes; Fig. 1B). The linear regression equations for TVP were y = 0.133 x + 15.122 on the right eyes and y = 0.065 x + 18.343 on the left eyes where y = TVP and x = age, and for TPA y = 0.195 x + 13.747 on the right eyes and y = 0.145 x + 16.230 on the left eyes where y = TPA and x = age, respectively.

**Figure 2:**
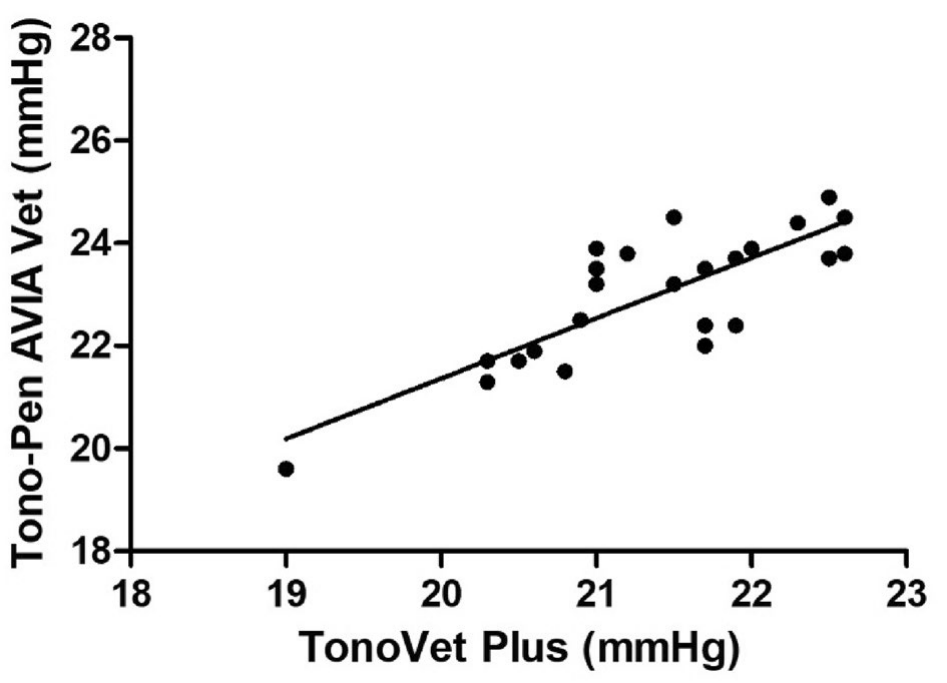
Scatterplots and linear regression models showing correlation between the mean intraocular pressures measured with Tono-Pen AVIA Vet and TonoVet Plus for post-pubescent Dutch belted rabbits.

**Figure 3:**
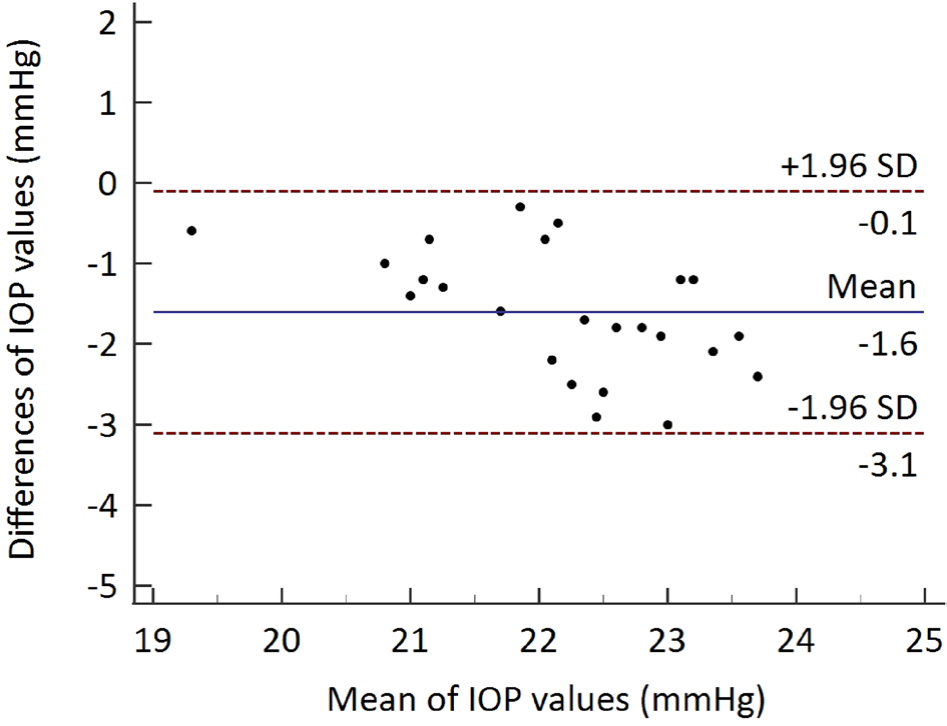
Bland–Altman plots comparing the mean intraocular pressure (IOP) measurements obtained with Tono-Pen AVIA Vet and TonoVet Plus in post-pubescent Dutch belted rabbits. The solid line represents the mean difference, while the dotted lines indicate the upper and lower 95% limits of agreement.

Equation y = 0.560 x + 8.506, where y = TVP and x = TPA (r = 0.81, *P* < 0.001; Fig. 2), was produced by the linear regression generated from the data acquired by TVP and TPA. The Bland–Altman plot demonstrated that the two devices were generally in agreement, with 95% limits of agreement of (− 1.927, − 1.281) in mmHg when subtracting TPA from TVP (Fig. 3). The findings indicated that, on average, the TVP recorded 1.6 mmHg lower values than the TPA (*P* < 0.001). The two tonometers’ intra-observer agreement was good, as reflected by the intraclass correlation coefficient (ICC) of 0.76 (95% confidence intervals: 0.518–0.890).

The slit-lamp biomicroscopy data revealed no cases of possible corneal complications after the IOP measurements employing topical anesthetics.

## Discussion

In this study, the IOP of Dutch belted rabbits was measured using two different tonometers (rebound tonometry and a Tonopen) over the course of 22 weeks. The rabbits’ IOP tended to increase significantly as they aged, and it was higher when measured by Tonopen than when measured by rebound tonometry.

Rabbits have been the subject of many ophthalmic investigations, owing to the similarities between the anatomy and physiology of the eyes of rabbits and those of humans^6,7^. Indeed, rabbit-based animal studies are essential to the development of new glaucoma treatment techniques. New Zealand white rabbits have frequently been employed in these investigations, and a great deal of research has been done on their characteristics^8–10^. Nonetheless, Dutch belted rabbits often are preferred for ocular studies, due to their pigmented irides. Male Dutch belted rabbits reach puberty between the ages of 12 and 28 weeks, according to estimates based on a previous study in which testosterone levels began to rise at 10 weeks and dropped at 12 weeks^3^. At about 28 weeks of age, meanwhile, the serum testosterone level stabilized and testicle growth stopped^3^. Age-related IOP alterations and variations in the IOP tonometry of Dutch belted rabbits, however, are not well understood. To appropriately interpret experimental results, defining IOP reference ranges is crucial. Thus, this study evaluated two widely used veterinary tonometers in an effort to better understand how IOP changes with age in Dutch belted rabbits.

In the results, IOP was observed to be positively correlated with age, which is consistent with several findings on age-related IOP changes in humans^11–13^. The mechanisms for age-related variation are uncertain, because the influences of age and ocular parameters on IOP are complex and cannot be studied in isolation. Zhang et al. studied variations in the corneal parameters of New Zealand white rabbits in relation to age, and revealed that both corneal hysteresis and corneal resistance factor are negatively correlated with age while central corneal thickness is positively correlated with age^5^. These observable age-related changes in corneal characteristics and aqueous humor dynamics may be partially attributed to the maturation and growth processes of the eye, which may have an impact on age-related IOP change. Similarly to this study, some previous investigations have determined that IOP rises with age in rabbits^10,14^. However, other studies have recorded various outcomes for rabbits of various ages^5^, and even in humans, there is conflicting evidence on the relationship between IOP and age when taking into account racial variances and other demographic characteristics^15,16^. Therefore, further longitudinal study of IOP and its relation to rabbit age is required.

TonoVet Plus (TVP) and Tono-Pen AVIA Vet (TPA) are two popular portable and non-invasive tonometers for animals. TVP utilizes rebound tonometry and is equipped with a magnetic probe. This probe is electromagnetically propelled against the cornea, resulting in a rebound from the cornea that induces a voltage change. This change is then converted into an electric signal, representing the IOP^17^. On the other hand, TPA is based on applanation tonometry and is widely employed across different species. It indirectly measures IOP by applying force to flatten a specific area of the corneal surface, and the resulting pressure reading is equivalent to the pressure in the eye^18^. According to the investigation of Ma et al., TVP and TPA both had outstanding intrasession repeatability and inter-operator reproducibility^19^. They did, however, also demonstrate that both tonometers had biases and underestimated manometric IOP. The present study determined that there was a difference in IOP measurements between TPA and TVP at various time points, the TPA measurements showing higher values. This outcome is comparable to that of previous research on New Zealand white rabbits^20^. These results might be related to the possibility of TPA overestimation, as this mode of measurement irritates eyes even after anesthesia with eye drops containing 0.5% proparacaine and also to the different internal calibration scales of those two devices. It is necessary to employ caution when interpreting such results since, despite the numerical difference between the two devices being statistically significant, it is impractical to take it as a clinically significant value.

There are several limitations to this study. First, relatively young post-pubescent rabbits were employed as study subjects. Second, only male animals were included in this study. Third, the follow-up period was brief, though it was still rather long compared with previous studies. Fourth, seasonal differences were not taken into account in this investigation. Nevertheless, the IOP-increase effect in the winter was lessened, because the data were collected throughout the spring and summer seasons. Fifth, we utilized only TPA and TVP in this experiment, though use of a hand-held Goldmann applanation tonometer (GAT) would have been suitable for comparison with both the TPA and TVP data, respectively, in a more comprehensive analysis. In fact, further research using the GAT may prove instructive for rabbit eyes, as a previous study showed a substantial difference between TPA and GAT IOP in primary congenital glaucoma^21^. Sixth, IOP measurements were the only ocular variables analyzed in this investigation, and it remains necessary to conduct additional research on variables including axial length and corneal pachymetry. Seventh and finally, the drawback is that alterations in IOP with aging might not have a major impact on clinical outcomes. If the follow-up time were longer and more subjects were enrolled, the outcomes might have been different. Because this study investigated changes solely in the IOP of male Dutch belted rabbits that had reached puberty, care must be employed when interpreting its findings.

This study found that IOP in post-pubescent Dutch belted rabbits tended to increase with age, and that a Tonopen consistently measured higher IOP values relative to rebound tonometry throughout the 22 week observation period.

## Methods

### Animals

Post-pubescent male Dutch belted rabbits aged 36 weeks and weighing 2.0–2.5 kg were obtained (Kitayama Labes Co. Ltd., Japan) and used in this study (n = 10 animals). The study procedures were approved by the Institutional Animal Care and Use Committee at Seoul National University Hospital (No. 21-0111) and conducted in accordance with the Association for Research in Vision and Ophthalmology Guide for the Care and Use of Laboratory Animals and ARRIVE guidelines (Animal Research: Reporting of In Vivo Experiments). The rabbits were housed in the climate-controlled vivarium of the Institute for Experimental Animals at Seoul National University under 12 h light and dark cycles. Food and water were available ad libitum.

### IOP measurements

IOP measurements were performed between March and August of 2023 for both eyes of each rabbit using both a rebound tonometer (TonoVet Plus; TVP, Icare, Finland) and a Tonopen (Tono-Pen AVIA Vet; TPA, Reichert Inc., USA) every 2 weeks for 22 weeks. After each tonometer was calibrated, the initial IOP measurement was performed. In each evaluation, TVP readings were taken first without topical anesthetic, and then, after a 5 min break, Tonopen IOP measurement was carried out using the TPA equipped with an Ocu-film tip-cover, the eyes having first been anesthetized with 0.5% proparacaine. TPA IOP measurement was performed latterly, due to what otherwise would have been the probable effect of topical anesthesia on the TVP employed in the rebound tonometry. The rabbits were placed on an examination table without the use of sedatives or calming agents, using only gentle manual restraint over the lower back region. To minimize stress during transport, the readings were conducted on-site, consistently by the same examiner (Y.I.S.). IOP measurements were taken with minimal pressure applied to the eyelids, just sufficient to keep them open. The measurements were performed in both eyes of each animal, randomly chosen with the aid of computer. The TPA has a measurement range of 1–99 mmHg, the device displaying individual readings and the average of 6 continuous readings along with a statistical confidence indicator. A repeat measurement was performed if the TPA’s statistical confidence indicator was less than 95%. The TVP has a measurement range of 1–99 mmHg as well, each IOP reading being obtained by subtracting the highest and lowest of 6 continuous readings and automatically averaging the remaining 4 readings. The species option for the TVP was set to “rabbits”. Measurements were made 3 times on each animal, and the average of 3 independent IOP measurements was calculated. The measurements were carried out repeatedly until the coefficient of variation was below 5%. IOP was measured under constant lighting conditions at about the same time every day (10–11 am) to minimize the impact of diurnal variation. To examine any possible corneal complications, slit-lamp biomicroscopy was performed immediately following IOP measurement.

### Statistical analyses

The IOP values are presented herein as means with standard deviations. The normal distribution of data was evaluated using the Kolmogorov–Smirnov test. The average IOP differences between TVP and TPA were compared using a two-tailed paired Student’s t-test, and Bland–Altman analysis was used to assess the agreement between two distinct tonometers. Repeated-measures ANOVA tests were conducted to evaluate IOP changes of the right and left eyes over time. The IOP measurements acquired by TVP and TPA were displayed in scatter plots and simple linear regressions were conducted to estimate their association. To determine the degree of correlation between the two tonometers, Pairwise Pearson’s correlation coefficients were computed for the IOP values. The intraclass correlation coefficient (ICC) was used to measure the agreement between the two tonometers in their IOP measurements. The associations between age and measured IOP were also evaluated using Pairwise Pearson's correlation coefficients and linear regression models. The criterion for statistical significance was determined to be a *P* value less than 0.05. Statistical analyses were performed using SPSS software (version 25.0; IBM Corporation, Armonk, NY, USA).

### Ethical approval

Animals were handled in an ethical manner without using excessive force or causing them discomfort. The study procedures were approved by the Institutional Animal Care and Use Committee at Seoul National University Hospital (No. 21-0111) and conducted in accordance with the Association for Research in Vision and Ophthalmology Guide for the Care and Use of Laboratory Animals.

## Data Availability

The authors are willing to provide, upon reasonable request, the datasets produced during the current investigation by the corresponding author (KH Park).

## Abbreviations

IOP: Intraocular pressure
TVP: TonoVet Plus
TPA: Tono-Pen AVIA Vet
GAT: Goldmann applanation tonometer
ICC: Intraclass correlation coefficient
